# Topics of Public Interest

**Published:** 1909-12

**Authors:** 


### TOPICS OF PUBLIC INTEREST.

---

### Plan to Save Children from Tuberculosis—Unique Institution Founded to Rescue Boys and Girls Who Have Been Infected with Disease—Restored to Normal Health in the Lakewood Pines.

---

With gifts aggregating $700,000 an absolutely unique institution, a Tuberculosis Preventorium for Children, was formally organized, November 9, 1909, at a meeting held at the residence of Henry Phipps, Fifth avenue and Eighty-Seventh street, New York. Its purpose is to take from the tenements children who have been infected with tuberculosis and restore them to normal health before it is too late. Among all the institutions for the care of the victims of the great white plague, there is none that offers escape from the disease to the child who is in the first stages and who can be cured.

This radical step in the fight against tuberculosis had its inception last May, and the Preventorium actually began its work on July 2, when the first child was received, but no inkling of the fact was given to the public until this afternoon, when Marcus M. Marks, the president, made the announcement that the institution was actually in successful operation.

He said the Preventorium had been founded to cope with the alarming condition revealed by the reporting of 23,325 new cases of tuberculosis in New York city in 1908 and by Dr. Herman M. Biggs's estimate that there were 40,000 children in the tenements who had been infected with the disease, most of whom could be saved by open air life, pure food and wise supervision.

### HALF MILLION GIFT FOR PREVENTORIUM.

Mr. Marks said:

The Tuberculosis Preventorium for Children is the first institution of its kind in this country. The work has been inspired and made possible by the far-sighted liberality of Mr. Nathan Straus, whose active efforts to reduce infant mortality have already made him known the world over. Mr. Straus has presented to us the Cleveland cottage and surrounding eight acres of pine woods at Lakewood, N. J., and a majority of stock in the Lakewood hotel property, in which his investment amounts to $500,000. There is no encumbrance or condition to this gift. We may either arrange to use the hotel property or sell it and use the proceeds in constructing around the Cleveland cottage such buildings as we may require.

Occupation of mind and body will second the good effects of the fresh, fragrant air of Lakewood, which, with its dry, porous soil, offers an ideal location for a Tuberculosis Preventorium. Practical instruction will be given in carpentering, cobbling, basketry, weaving, stencil work, metal work, and the like. Miss Dorothy Whitney has munificently endowed this department by a gift of $100,000, the interest of which will pay for instructors, tools and materials.

We have had substantial voluntary cash donations toward our running expenses from Mr. Henry Phipps, Mr. Isaac N. Seligman, Mr. Jacob Wertheim, Mrs. Walter B. James and Mr. Jacob H. Schiff in advance of our first appeal for funds.

### NINETY-TWO CHILDREN RECEIVED.

Possession was taken of the Grover Cleveland cottage in May, and it was readily adapted, under the personal direction of Mrs. Cleveland, the porch being arranged for six beds and the house for fourteen, these quarters being for girls, and an open air camp about 100 feet long was built to accommodate twenty boys.

With the assistance of Dr. James Alexander Miller an arrangement was made with the New York Association of Tuberculosis Clinics for the selection of children, and the first patient was received on July 2. Since then ninety-two children, ranging from four to fourteen years, have been received from thirteen clinics or other institutions, and all have shown steady improvement, all gaining in weight, some eight or nine pounds, and the capacity of the institution has been so taxed that it is hoped to make room for 400 children by next summer.

The work has shown that children slightly affected with tuberculosis, when brought within the benefits of the pine air of Lakewood and the regimen of the Preventorium, may often be completely restored to normal health within three months.

### RESULTS SHOWN BY TYPICAL CASES.

Dr. Alfred F. Hess has complete charge of the work, with advice from Dr. Abraham Jacobi, Dr. Herman M. Biggs and Dr. S. S. Goldwater. Mrs. Ella Wheelwright is chief nurse; Mrs. Mary Morgan, matron, and Mrs. Anne Thompson, for seventeen years nurse in the Cleveland family, is one of the nurses. Mr. Wheelwright, an expert in industrial training, keeps the boys employed in practical handiwork.

With this staff the work has been practically demonstrated, as shown by these typical cases:

CASE A—Boy, aged ten. Father, mother, two brothers, tuberculous; home conditions poor; family supported by charities; tuberculin test on the boy positive; not yet in infectious stage; gained seven pounds at Preventorium and returned home perfectly well; whole family then moved to Liberty, N. Y.

CASE B—Girl, aged twelve. Mother, brother, sister, tuberculous; home conditions poor; tuberculin test on girl positive; not yet in infectious stage; gained four and one-half pounds at Preventorium. Little brother and sister since received as patients.

### SAVING DOOMED CHILDREN.

These children would have been doomed to suffering and early death left in their homes. Taken to Lakewood, which Dr. Loomis described as the most ideal place in the world for tuberculous patients, they were saved.

The plan includes systematic improvement of their home conditions, while the children are in the Preventorium, and careful "follow-up" work, in coöperation with the tuberculosis clinics, so that the benefits of the Preventorium may be permanent.

It is expected in this way to save many children from the life of suffering that is the lot of the tuberculous patient, and to reduce the demands upon the sanitoria that are the only hope of advanced cases.

### NO EXPENSE TO PARENTS.

In order that this work may be readily efficacious in stemming the plague by reaching the poorest children, it is planned to maintain the Preventorium without charge to parents for board or railroad fare. To care for 400 children will cost about $100,000 a year. The trustees, therefore, seek an endowment of $1,000,000 which would cover 40 per cent. of the running expenses and assure the permanence of the work.

A gift of $2,500 will permanently endow a bed; $50,000 will endow an open-air camp of twenty-four beds, with nurse's room, shower and bath.

For the $60,000 additional needed after the endowment of a million is secured, the Preventorium will depend upon membership fees, in sums ranging from $1.00 to $1,000, checks for which may be sent to Mr. Alexander S. Webb, Jr., treasurer, care the Lincoln Trust Co., Madison Square, or to the president, Marcus M. Marks, 687 Broadway.

BOARD OF TRUSTEES.

The management of the institution is in the hands of the following board of trustees:—

Marcus M. Marks, president; Mrs. J. Borden Harriman, first vice-president; Mr. Henry Phipps, second vice-president; Mr. Isaac N. Seligman, second vice-president; Miss Dorothy Whitney, secretary; Alexander S. Webb, Jr., treasurer; Archibald S. Alexander, Dr. Herman M. Biggs, Mrs. Grover Cleveland, R. Fulton Cutting, Dr. Alfred F. Hess, John S. Huyler, Miss Mary Harriman, Dr. Abraham Jacobi, Mrs. W. B. James, Arthur C. James, W. G. McAdoo, Dr. James Alexander Miller, Eugene Meyer, Jr., Thomas M. Mulry, Celestino Piva, Jacob A. Riis, M. Theodor Rosenberg, Jacob H. Schiff, James Speyer, Henry L. Stoddard, Lawrence Veiller, Mrs. Mary Hatch Willard, Felix M. Warburg.

